# Dispositions that matter: Investigating criminalized women’s resettlement through their (trans)carceral habitus

**DOI:** 10.1177/17488958211017371

**Published:** 2021-05-21

**Authors:** Kaitlyn Quinn

**Affiliations:** University of Toronto, Canada

**Keywords:** Bourdieu, carceral habitus, criminalized women, embodiment, resettlement, transcarceration

## Abstract

Whether prisoner resettlement is framed in terms of public health, safety, economic prudence, recidivism, social justice, or humanitarianism, it is difficult to overstate its importance. This article investigates women’s experiences exiting prison in Canada to deepen understandings of post-carceral trajectories and their implications. It combines feminist work on transcarceration and Bourdieusian theory with qualitative research undertaken in Canada to propose the *(trans)carceral habitus* as a theoretical innovation. This research illuminates the continuity of criminalized women’s marginalization before and beyond their imprisonment, the embodied nature of these experiences, and the adaptive dispositions that they have demonstrated and depended on throughout their lives. In doing so, this article extends criminological work on carceral habitus which has rarely considered the experiences of women. Implications for resettlement are discussed by tracing the impact of criminalized women’s (trans)carceral habitus (i.e. distrust, skepticism, vigilance about their environments and relationships) on their willingness to access support and services offered by resettlement organizations.

## Introduction

Whether prisoner resettlement is framed in terms of public health, safety, economic prudence, recidivism, social justice, or humanitarianism, it is difficult to overstate its importance. In Canada, more than 75% of all prisoners will eventually navigate this transition—for many, multiple times (Public Safety Canada, 2015). Intensifying calls to decarcerate amid the COVID-19 pandemic (Theodore, 2020) poses an increasingly urgent need for better understandings of how former prisoners experience resettlement. This is particularly true for women, who have historically received less attention in scholarship and policy discussions (Malloch and McIvor, 2011; Österman, 2018). Accordingly, this article investigates the experiences of women exiting prison in Canada to deepen understandings of women’s post-carceral trajectories and their implications.

Much of the existing research on resettlement and desistance has prioritized cognitive explanations (e.g. Giordano et al., 2002; Maruna, 2001) to the detriment of understandings of the embodied, habitual, and pre-conscious dimensions of exiting prison (Bosworth and Kaufman, 2013; Chamberlen, 2017). By contrast, in this article, I center embodiment in processes of resettlement in order to understand how punishment and its effects are absorbed and actually experienced by individuals *in* bodies. To do so, I draw on and extend the Bourdieusian concept of carceral habitus (e.g. Caputo-Levine, 2013; Page and Goodman, 2018). In particular, I place existing accounts of carceral habitus, which generalize from the experiences of criminalized men, in dialogue with feminist understandings of criminalized women’s marginalization as uniquely transcarceral, to offer the concept of *(trans)carceral habitus.* Analytically, this adaptation emphasizes the fluid entanglements of criminalized women’s marginalization within, beyond, and before prison; the embodied nature of these experiences; and the creativity, adaptations, and resilient dispositions criminalized women have demonstrated and depended on throughout their lives.

Greater knowledge and understanding of the embodied dispositions associated with criminalized women’s (trans)carceral habitus may also inform better resettlement support and service provision by assisting helping professionals anticipate the impact and interpretation of their rehabilitative interventions (e.g. Walklate, 2011), including at the pre-conscious level. Existing scholarship has illustrated how the post-prison dispositions of criminalized men can create barriers for their resettlement (e.g. Caputo-Levine, 2013; Martin, 2018). However, these outcomes have yet to be specified for women who face distinct pathways into and out of crime (Harding et al., 2019; Western, 2018). My analysis demonstrates how criminalized women’s formative experiences and transcarceral marginalization may encourage particular (adaptive) dispositions that reduce (or, at the very least, complicate) their ability and willingness to access resettlement services.

This article is structured as follows. I discuss recent scholarship on resettlement and desistance, highlighting the need for embodied perspectives. I introduce the concept of carceral habitus, placing it in dialogue with feminist criminological literature on transcarceration to better account for the resettlement realities of criminalized women. I define (trans)carceral habitus—this article’s theoretical contribution. I detail my data and methods. Subsequent empirical sections illustrate how criminalized women’s embodied dispositions throughout their resettlement connect to their (often) lifelong experiences of marginalization, social isolation, and threats of violence informed by and intersecting with their gender, race/ethnicity, and social class. Finally, I underscore the importance of these findings for studies of resettlement, explain how this research might inform rehabilitative interventions undertaken with criminalized women, and highlight study limitations and avenues for future research.

## Desistance versus resettlement

Resettlement refers to the transition individuals make from incarceration to communities (Travis, 2005; Western, 2018). One of the primary concerns in understanding prisoners’ return to communities is whether or not they re-offend (Harding et al., 2019). Theories of desistance highlight social bonds (Laub and Sampson, 2003), self-conceptions (Maruna, 2001), and the interplay between the two (Giordano et al., 2002; Healy, 2013; Leverentz, 2014) as individuals strive to reduce their involvement in crime. However, the focus on re-offending is quite narrow, methodologically ambiguous, and can be detrimental to understanding criminalized individuals’ experiences and ambitions *on their own terms* (Maidment, 2006). Accordingly, there has been a recent push to investigate the process of resettlement *in itself*, recognizing that “desisting from crime is just one component of successful reintegration” (Harding et al., 2019: 8).

Resettlement research, then, differs by centering the realities and perspectives of those who are “trying to find a place in society after incarceration” (Western, 2018) over concerns about re-offending. At the individual level, this research has focused on the “pains of release” (McKendy and Ricciardelli, 2020)—the challenges and barriers ex-prisoners face (e.g. poverty, homelessness, (mental) health, substance use, unemployment, relationships; Harding et al., 2019; Mann et al., 2019; Travis, 2005). At the cognitive level, research has illuminated how criminalized individuals understand themselves and their experiences amid these challenges (Healy, 2013; Stevens, 2012). And, at the community level, others have demonstrated how the effects of incarceration and resettlement are concentrated in certain (often poor and/or racialized) neighborhoods (Morenoff and Harding, 2014; Wacquant, 2001).

Within this rich literature, considerations of *embodiment* have largely, and problematically, been absent (Bosworth and Kaufman, 2013; Messerschmidt, 1999). Limited research has illuminated how incarceration becomes inscribed on (ex-)prisoners’ bodies through speech and dress (Halushka, 2016), self-injury (Chamberlen, 2017), missing teeth (Moran, 2012), and tattoos (Phillips, 2001). While these experiences are sometimes linked to post-prison outcomes (e.g. Caputo-Levine, 2013; Martin, 2018), the relationship between embodiment and resettlement remains significantly under-theorized (Moran, 2012). In the next section, I refer to the burgeoning literature on carceral habitus as a pathway forward in this regard.

## Carceral habitus and gender

Generally speaking, habitus refers to the enduring and embodied dispositions—for example, preferences, manners of standing, moving, speaking, and taking up space—that are informed by an individual’s structural position (Bourdieu, 1984). In the criminological literature, *carceral habitus* describes the “dispositions that shape conscious and preconscious practice within and beyond carceral institutions” (Page and Goodman, 2018: 7). This concept has predominantly been mobilized to describe the impact of incarceration on (ex-)prisoners’ habits, preferences, and embodied dispositions. In this article, the specific aim in mobilizing habitus to theorize criminalized women’s experiences is to underscore the relationship between the micro-details of their everyday (and embodied) realities and the structural constraints (e.g. social marginalization, patriarchy, racism, colonialism) within which they were often positioned. Rather than prioritizing either micro (agentic) or macro (structural) explanations of resettlement—a dichotomy that existing research has tended to entrench (Österman, 2018)—in using habitus, this article makes a theoretical commitment to understanding this process as multi-scalar and to investigating the interstices wherein these factors inevitably mix and mesh.

Gender has emerged as a *central* feature in this literature, with the majority of research illustrating how the hyper-masculinity of prison spaces encourages men’s continued violence upon release (e.g. Halushka, 2016; Page and Goodman, 2018). For example, Caputo-Levine (2013) uses carceral habitus to explain participants’ readiness to initiate violence on the outside, offering an example of a participant becoming overwhelmed in public and “almost flattening a woman who bumped into him” (p. 179). However, as Shammas (2018) asserts, “not all penal institutions are sites of extreme bodily danger” (pp. 212–213)—for example, prisons in Northern Europe—wherein such violent dispositions would be cultivated. Extending this critique, it is my contention that the carceral habitus, as currently specified, is poorly positioned to account for the experiences of criminalized women.

New theory building in this area requires a move away from predominantly male and American research samples to uncover and account for more diverse empirical realities. This article seeks to extend (and reorient) existing scholarship by centering women’s experiences of resettlement. The limited research using habitus to understand women’s experiences has focused on the stories criminalized women tell—via a “narrative habitus”—to reconcile criminal actions with conceptions of femininity (Fleetwood, 2016; Otto, 2020). My research, however, focuses firmly on the experiential realm and seeks to draw out *what resettlement is like* for women from an embodied perspective.

## Women’s transcarceral trajectories

There is scholarly consensus that women’s experiences of criminality, incarceration, and resettlement are “so distinct” (Western, 2018: 8) as to warrant separate consideration (Harding et al., 2019; Österman, 2018). Yet, women’s experiences of the criminal justice system, and release from prison in particular, have received significantly less attention than men’s experiences (McKendy and Ricciardelli, 2020; Pollack, 2009). Feminist criminologists have made substantial contributions to resettlement research in recent decades (Barberet, 2014; Österman, 2018), placing gendered realities at the center of their research agendas. Scholars have demonstrated how women’s marginalized positions at “the fringes of communities, the state, gender propriety and the criminal justice systems” (Baldry, 2010: 260–261) have accompanied clusters of “troubling” features in their life histories (Western, 2018: 8). When compared with men, criminalized women disproportionately experience poverty, drug use, trauma, homelessness, violent and sexual victimization, and poor physical and mental health, in addition to assuming (or trying to regain) primary caretaking responsibilities for children (Leverentz, 2014; Österman, 2018; Pollack, 2009).

When accounting for such realities, feminist scholars often invoke the term *transcarceration* to signal the continuity of women’s control and punishment across state and social institutions (e.g. peno-juridical, education, health, welfare; Lowman et al., 1987). The transcarceral paradigm allows scholars to account for the “long-standing and layered experiences” of punishment and control that criminalized women navigate *prior* to their incarceration (Maidment, 2006: 74). In doing so, transcarceral accounts actively resist characterizing incarceration as “the beginning of a narrative on punishment” (Chamberlen, 2017: 130) and instead press for broader, richer accounts of women’s marginalization that detail how their experiences are structured by patriarchal, racist, colonial, and capitalist systems of oppression that both precede and extend beyond imprisonment (e.g. Carlton and Baldry, 2013; Maidment, 2006; Pollack, 2009). However, in accounting for such breadth, much of the existing research has prioritized linkages across systems and structures at the expense of understanding how these connections are actually experienced and embodied.

The *(trans)carceral habitus*, then, seeks to account for the constellation of embodied and adaptive dispositions accrued throughout criminalized women’s experiences of marginalization and social control within, beyond, and prior to their imprisonment. This theoretical innovation also serves to disrupt the priority that much of the Bourdieusian criminological work has granted to the “moment” of transition between prison and community, wherein ex-prisoners experience a “mismatch between dispositions [developed in prison] and expectations [of the community]” (Martin, 2018: 674). Dispositions forged in prisons can endure upon release, but crucially also include lived and embodied experiences prior to incarceration. By focusing on the *continuities* of criminalized women’s experiences across their lifespan, rather than only the differences brought to bear by imprisonment, this article offers a richer and more expansive understanding of ex-prisoners’ embodied adaptations to long-standing social marginalization as they navigate resettlement.

## Data and methods

This article uses data from a wider research project undertaken with university ethics approval between 2013 and 2015.^1^ The analysis that follows draws on fieldnotes collected during hundreds of hours of participant observation with 21 formerly incarcerated women and 16 interviews conducted with eight women (see Table 1 for demographics). None of the participants were currently on parole or probation. Participants were recruited using connections I developed as a volunteer working with criminalized women.^2^ Fieldwork consisted of shadowing participants for approximately 2–3 days per week for 6 months, visiting their homes, accompanying them on errands (e.g. visiting food banks, doctor’s offices), sharing meals, and visiting their places of work. Participant observation was also conducted in three voluntary organizations offering resettlement services to criminalized women (hereafter referred to as resettlement organizations) where I informally spoke with staff and volunteers, participated in programming, and spent time with criminalized women during drop-in hours. Resettlement organizations undertake a variety of “prisoner incorporation” (Kaufman, 2015) functions including providing employment counseling, housing support, drug/alcohol treatment, and a variety of “soft” skills programming. In Canada, these organizations are typically non-profits or registered charities that receive a good proportion of their funding from the local, provincial, and federal governments. Larger organizations often also have service delivery contracts with Correctional Service Canada to run court mandated or criminal justice diversion programming (e.g. halfway houses, anger management, domestic violence; Quinn, 2020).

**Table 1.** Participant demographics.

*n* = 21	
Place of birth	
Canada	16
Another country	5
Age	
20–29	11
30–39	4
40–49	5
50+	1
Race/ethnicity (self-identified)	
White	15
Asian Canadian	2
Indigenous	4
Education	
Less than high school	8
High school diploma	4
Some university	4
University degree	0
Unknown	5
Partner/spouse	
Yes	6
No	15
Children under 18	
Yes, with contact	4
Yes, no contact	5
No	12

Observations were triangulated by two semi-structured in-depth interviews conducted with 8 of the 21 women I observed. I began with a list of themes (i.e. participants’ social ties, experiences of exiting prison, daily activities, experiences with resettlement programming, and their hopes and expectations for the future) and prepared questions on each topic, permitting room for digressions. Interviews lasted between 42 and 110 minutes, were tape recorded, and transcribed. Participants determined the time and place of interviews, typically coffee shops, public outdoor spaces, or their homes. All participants gave informed consent and pseudonyms are used throughout.

Thematic analysis was completed in NVivo by generating themes inductively through open, axial, and selective coding (Strauss and Corbin, 1998). During open coding, I read through the entirety of my data creating working descriptions of what I saw happening. Beginning with 36 unique codes, I fleshed out the relationships between these descriptions, seeking to make connections between what women told me about their social relationships as they exited prison and their larger experiences of marginalization. This process produced two topical categories: social isolation and safety. Throughout this iterative process, and in conversation with the literature, the core idea of (trans)carceral habitus emerged. To refine this idea, I re-read and re-coded my data selectively through this lens. In the two sections that follow, I mobilize short narratives from my fieldnotes, excerpts from interviews, and analytical guidance from other scholars to operationalize criminalized women’s (trans)carceral habitus and its implications for their resettlement.

## Embodying experiences of social isolation

Despite a wealth of multi-disciplinary literature that demonstrates the importance of social relationships in a variety of positive post-prison outcomes (e.g. Harding et al., 2019; O’Brien, 2001), participants in this research struggled to form connections with others. They frequently felt lonely, unsupported, or dissatisfied with their past and/or current social ties. The women included in this research also displayed a cluster of embodied dispositions toward their relationships—that is, distrust and skepticism, relational vigilance, and resistance toward forming social ties. In this section, I demonstrate the relationship between ongoing experiences of social isolation, habitus, and criminalized women’s experiences of resettlement.

Mik got up from the crumbling concrete wall we were squatting on in the alleyway, kicking pebbles downhill. “I don’t have anyone. Basically, everybody that I knew just stopped talking to me. They didn’t want anything to do with me,” she said, bending down to examine a scuff on her shoe. Mik is an Indigenous woman in her mid-20s who lost her family at a young age and spent the majority of her youth and adolescence moving between foster homes. She abruptly left her last placement when she was 17. “I just had to get away from there,” she explained. Mik reached out to a number of voluntary organizations, but found herself awkwardly caught between those that helped children and those that only helped adults. “It’s just me,” she said, emphatically thumping her breastbone. “Same bullshit as always. I don’t reach out like that anymore. Problem solved.”

When social ties are invoked in criminological research, they are generally coupled with declarations of the (emotional and material) resources these connections provide throughout resettlement (e.g. Giordano et al., 2002; Laub and Sampson, 2003). In contrast to the pro-social effect that family and intimate relationships can have for men (Markson et al., 2015; Maruna, 2001), this is often not true for women (Leverentz, 2014; McKendy and Ricciardelli, 2020). However, my data indicate that the absence of pro-social resources is only *one* of the implications low social capital can have on women’s experiences of resettlement. Enduring experiences of marginalization and isolation can also generate a set of embodied dispositions that exercise a determinative impact on women’s resettlement *separate from* existing explanations of social capital.

For example, in interpreting and internalizing her life experiences, Mik gained an intuitive “feel for the game” (Bourdieu, 1998: 25), wherein others tended to treat her poorly or reject her. She adapted by quickly establishing others as the enemy—eager to reject them before they had the opportunity to confirm her suspicions.^3^ She rarely made eye contact and, instead, typically dragged her feet in patterns on the ground while others were talking. I often observed her pretending not to hear others calling out “hello,” and quickly change directions. Mik’s habitus, then, involved a cluster of strategies to create physical (and thus emotional) space between herself and others. Outside of valid arguments about her lack of social capital, these dispositions that had been forged throughout her life—and in particular, their embodied expression—created barriers for her resettlement.

For example, on one occasion, Mik was making coffee at a drop-in center for women when a volunteer approached from behind and asked if she needed anything. Startled, Mik knocked over her cup as she fumbled to fit the lid. “Not from you I don’t.” Visibly distressed and disoriented, she quickly made for the door, leaving her cup rolling around on the wet floor. When I caught up with her later, she explained that in her rush to leave she had forgotten her bag and it was now missing. “If it wasn’t for that woman, I wouldn’t be in any of this mess.” Mik told me she would not be completing the resettlement program she was enrolled in at this organization, claiming, “I was better off on my own . . . as usual.” Beyond this specific instance, Mik’s response drew on a lifetime of examples in which “helping” interventions were experienced as harmful. Her time in the foster system, though ostensibly for her benefit and safety, was painful, scary, and disappointing—an experience common for many Indigenous children and young people in care (Fournier and Crey, 1998). Indeed, in Canada such (false) rhetoric of “helping” has long been used as a settler-colonial justification for violent “civilizing” interventions undertaken in the lives of Indigenous Peoples (Bell and Schreiner, 2018). Mik’s short time at the drop-in center—and, in particular, the interaction recounted here—served to harden her existing dispositions (i.e. resistance, skepticism) toward relationships. Her experiences demonstrate that the sensory overload and social disorientation that other researchers have attributed to ex-prisoners’ geographic transition from rural prisons to urban centers (e.g. McKendy and Ricciardelli, 2020) can also be experienced because of embodied adaptations to social interactions that pre-date their imprisonment.

Courtney is a white woman in her late 20s who demonstrated a similar cluster of embodied dispositions as she navigated her resettlement. At times she seemed enthusiastic to share and connect, willingly bringing up her vulnerabilities, but the door was quick to swing shut. Courtney told me that she was hospitalized when she attempted to take her own life. Obviously uncomfortable, she got up and disappeared into her bedroom. “I’m done now” she yelled from behind the door. In interactions with myself and others, I frequently observed Courtney avoid closeness and sustained vulnerability. She laughed after making emotional disclosures, quickly changed the subject, or, as demonstrated in this example, left abruptly and without explanation.

Although Courtney was vigilant in her interactions with others and wary of closeness, at times she appeared desperate to connect. She often blurted out her feelings, sharing more in the moment than she was ultimately comfortable with, and then quickly recoiling. Like Mik, these dispositions created barriers for Courtney’s resettlement. Her relationships suffered ongoing tension as she fought with her own comfort level. Not only did Courtney laugh as a coping mechanism when she felt vulnerable, but she also did so when listening to the disclosures of others. This latter disposition posed ongoing problems in her resettlement support group. Others expressed their frustration to me, and many eventually pulled back their support—which only served to validate Courtney’s cautious and skeptical disposition toward others. Here, we see evidence of the habitus’ inertia; it is structured *by* past marginalization and also structuring *of* future experiences (Bourdieu, 1984). Although Courtney’s experiences of incarceration undoubtedly influenced how she now relates to others (e.g. Caputo-Levine, 2013; Halushka, 2016), in talking about her resettlement she more often referenced the impact of factors that long pre-dated her criminal justice involvement (e.g. mental illness, strained family relationships, family violence, poverty) lending support to this article’s transcarceral view.

As a final example, I will draw on the perspective of Ofelia, an Asian Canadian woman in her 40s, who had been able to secure a close and affirming friendship with a prison volunteer named Sarah, despite demonstrating a similar habitus to Mik and Courtney. In getting to know Ofelia, she explained how her experiences of partner violence and other betrayals in her romantic and family relationships made her skeptical of others, even before her incarceration: “you don’t trust no one right away . . . you gotta worry about everyone.” Despite this long-standing disposition, Ofelia was able to connect with and eventually trust Sarah, whom she continues to rely on. The following excerpt illustrates how and why she experienced this relationship differently.

Um . . . yeah . . . Alright so for some reason I missed a couple of meetings [with Sarah]. I don’t remember why. I could’ve been in trouble or maybe I just didn’t feel like coming out [. . .] When I finally showed up, she was real pissed. She told me that if I was gonna miss another meeting then she was gone. She drives a long way, so she said she just wasn’t gonna do it anymore if I wasn’t going to show up [. . .] I don’t think I responded real well. I don’t remember what I said or anything but I remember her saying something like to think on it and let her know if I was in or out next time she came [. . .] I was pissed . . . But then I don’t know I guess I realized it was kind of nice. Like to have someone care enough to get mad if you don’t show up.

Ofelia’s experiences, in particular, offer important insights for how criminalized women might be better supported in their resettlement through attention to their habitus. This process of initial vigilance and skepticism, a phase of relationship “testing,” and (perhaps) eventual trust was especially reflective of participants’ relationships with volunteers and voluntary sector practitioners offering resettlement programs and services. In practice, this “testing” phase involved things like calling staff/volunteers in the middle of the night to see if they would answer the phone, showing up hours late to meetings, initially rejecting help from others, and telling particular stories (or using certain language) for their intended shock value and the distance this was thought to create. However, as Ofelia’s perspective demonstrates, habitus is not strictly determined. It can adjust (albeit slowly) to changing conditions and choices.

The examples I have shared from Mik, Courtney, and Ofelia’s experiences resonate with existing research on women’s resettlement and social relationships, supporting the “relational isolation” (Jones, 2009) or “relationship avoidance” (Leverentz, 2014) that others have observed. My research extends these concepts by showing how some women avoid both platonic and romantic relationships as an adaptive, embodied, and habitual disposition to eschew further disappointment and instability, rather than as a strategy to prevent re-offending as existing accounts have suggested. By utilizing the theoretical lens of (trans)carceral habitus, participants’ skepticism, vigilance, and/or resistance toward social ties were revealed as embodied, habitual, and pre-conscious occurrences, rather than strictly cognitive or strategic choices as the resettlement literature has dominantly presumed. In particular, I have demonstrated that criminalized women’s transcarceral and embodied experiences of social marginalization and isolation—often preceding their criminal justice involvement—have significant implications for their willingness to participate in resettlement services and programming.

Although this section has highlighted criminalized women’s experiences of isolation and vigilance, this is not to say that criminalized men do not share some of these experiences. Within their relationships, criminalized men are portrayed as wary of showing “any softness, any weakness,” often carrying themselves in a specific manner as to “announce to others that [they] can defend [themselves] if necessary” (Caputo-Levine, 2013: 172, 175). Men’s relational vigilance is disproportionately oriented toward conflict and physical aggressiveness (Page and Goodman, 2018). By contrast, for the women included in this research, their vigilance was primarily reflected in their desire to avoid disappointment by tempering their expectations of relationships, isolating themselves from others physically and emotionally, and underscoring their preference for being on their own over being vulnerable to rejections/disappointments from others. Although participants faced threats of physical violence, none mentioned using anticipatory or retaliatory violence in ways that matched existing descriptions of men’s carceral habitus (but, see Jones, 2009; Otto, 2020). And, in contrast to research examining criminalized men’s social isolation as a symptom of the “shock of release”—that is, their post-prison isolation is located in the broken or absent connections while incarcerated and/or the stigma of a criminal record (Durnescu, 2021; Martin, 2018)—the isolation described by women included in this research had much longer, transcarceral origins. In the next section, I turn to criminalized women’s experiences navigating unsafe environments and interactions, the embodied dispositions they cultivated in response, and the implications of this habitus for their resettlement.

## Embodying concerns about safety

In addition to experiences of social isolation, persistent threats to their safety and the limited resources criminalized women could draw on to protect themselves from violence also structured their resettlement (Jones, 2009; Miller, 2008). In this research, these concerns impacted *where* participants lived and visited, *whom* they saw and loved, and oftentimes quite literally *how* they moved.

Brooke walked at a stunning pace whenever we were together. She darted effortlessly between pedestrian traffic as we approached Tim Hortons (fast food restaurant) for a coffee and to warm up. The street was browned with December slush and oversized boots stomped and scratched over scattered salt—a harsh and discordant winter symphony. “Uhhh, ooop, sorry,” I muttered clumsily to the oncoming blurs. I skipped awkwardly every few steps to keep pace. Brooke’s head whipped around at every passing car. I found myself copying her. “What are you looking at?” She shrugged, “Habit, I guess.” Brooke, a White woman in her 30s, sells sex on most weekends along “the Avenue,” where I have seen her exhibit a similar vigilance over cars and pedestrians. At the beginning of my fieldwork, and for the majority of Brooke’s working life, selling sex was illegal in Canada. As a result, she instinctively scanned and moved quickly through her environments, feeling that her physical safety and desire to avoid arrest were “up to her.” She operated under the assumption—and suspicion—that those she met were police, or otherwise ill-intentioned.

After the passing of Bill C-36 in 2014, which decriminalized selling sex, police attention was reoriented toward purchasers. Resulting changes in client preferences (e.g. not providing their names, refusing to communicate ahead of time, wanting to drive to isolated environments, and rushing screening processes) served to reduce Brooke’s safety. “It’s just like ‘okay get in, hurry up’, really rushing you and in a split-second I have to make a decision.” She took off her gloves and pulled out a lighter from inside her jacket, flicking her thumb over the top. “You’re taking a chance when you get into different cars. You really don’t know if you’ll come back alive,” she said shifting her weight. “Sometimes I was sure I was coming home in a bag.” Brooke’s habitus, then, involved physical vigilance and (quickly) interpreting the body language and intentions of others and generalizing—sometimes problematically—from bad experiences. She explained, “I don’t do Black guys anymore because I had a bad experience.” Assuming the ill-intentions of others and widely applying lessons from unsafe experiences was a protective strategy. “It’s better to be wrong by being too cautious, than the other way around.” Brooke’s numerous periods of resettlement, then, have played out like a game of keep away—avoiding the gaze and grips of those with the power to hurt or imprison her. “I’m always looking for an exit . . . like can I get out of this car, which direction do I run, stuff like that.”

Brooke’s suspicions of others, though keeping her safe, have also discouraged her from accessing resettlement services. “Those places, they ask too many questions . . . I’m not trying to have any of that written down for the cops to barge in and all of a sudden have everything just handed over to them.” This perspective supports Pollack’s (2009) claim that the information gathering and documentation practices of resettlement organizations can contribute to the sense that their clients are “always being watched and assessed” (p. 89). When Brooke did access resettlement services, her approach mirrored her dispositions on the street: quick and vigilant. “She never sticks around here for long,” a volunteer recounted. “It’s a shame because we don’t know too much about her so we don’t really know what she needs from us.” For Brooke, the helping “gaze” reminded her of other punitive or violent gazes she more frequently experienced during and prior to her incarceration, and thus reduced her willingness to engage in resettlement support programming.

Another participant, a white woman in her 20s named Zoe, explained that the majority of her day is spent trying to ascertain how she will protect herself overnight. “It’s not safe for women to sleep outside alone,” she said as she flipped through the clothing on the rack in front of her. We were in a small basement room of a resettlement organization that, among other things, collects clothes for women exiting prison. “You are a walking target,” Zoe says pulling a sweatshirt over her head. “I hardly know any homeless woman who hasn’t been raped, hasn’t been beat on,” her voice muffled from inside the fabric. “You have to buddy up. Have a relationship . . . but then there are expectations.” Pulling off the sweatshirt in a move that leaves her hair crackling, she quipped, “Sex. Blowjobs.”

Here, Zoe mobilizes what Miller (2008) refers to as the strategic use of others (often men) for protection at night, as she has done for the majority of her adult life. Although utilized by many of the women included in this research, these strategies were not endorsed by any of the resettlement organizations where Zoe, or others, accessed services. In fact, these adaptations were actively discouraged. “They’re always trying to get me in a shelter instead . . . but it fucks with the rhythm of my day, you know?” Here, Zoe offers a critique of shelters’ occupancy policies (i.e. vacating each morning, lack of storage, lining up each evening) that matches the experiences described by others (e.g. Stuart, 2016). “Every time I go in [to resettlement organizations] I’ve gotta be pestered about where I’m staying and have this whole conversation about my choices again and again . . . some days I’m just not up for it,” she said shaking her head. “There are easier ways to get my hands on a sweater, you know?” Zoe preferred doing things “her way” (i.e. further criminality, mobilizing adaptations that sometimes place her in unsafe scenarios) to fielding the shaming and stigmatizing questions involved in accessing some resettlement services. This example, in particular, illustrates the overlapping care and control functions of resettlement organizations (Maidment, 2006; Quinn, 2020) and highlights the negative implications for criminalized women’s trust in, and thus willingness to access, services incorporating such elements of “control talk” (Pollack, 2009).

Concerns about safety were compounded for the Indigenous women included in this research. Because of generations of colonization, Indigenous women are often thought to “belong to space[s] in which violence routinely occurs and to have [bodies] that [are] routinely violated” (Razack, 2000: 93). In fact, 3329 Indigenous women have been documented as missing and murdered in Canada between 1946 and 2013 (Pearce, 2013). Layla, an Indigenous woman in her 20s, described what these realities have meant for her daily life across her pre- and post-prison experiences.

If you look Aboriginal and there’s johns in the neighborhood looking for somebody to exploit quite often they will target you [. . .] so you can’t even walk to the store without being harassed by a john. I was walking with my sister and I was pushing my nephew—he was two, he was still in his stroller. We were just coming back from the store . . . Middle of the day. And this one john just kept circling and circling and circling.

In response to such instances across her lifespan, Layla learned to embody particular protective dispositions as she navigates public spaces. “Get in, get out, no small talk, just do everything as quickly as possible and don’t ever make any eye contact with men.” However, because the threats and harassment Layla and other Indigenous participants regularly experience extend to scenarios they cannot avoid (e.g. getting groceries, taking public transit) the “situational avoidance” (Jones, 2009) utilized by other participants (e.g. Brooke’s decisions about clients and Zoe’s preferences about where to stay) were reliably unsuccessful.

When I asked her about accessing resettlement services, Layla replied, “Not really lately. I’m too damn scared to go over there anymore.” Here, she was referring to a recent experience of harassment in the resettlement organization’s neighborhood. “I’ve been staying well away from there these days.” As is the case in many cities, the social services that may be helpful (e.g. temporary employment agencies, food banks, resettlement organizations) are geographically concentrated in areas of high poverty, homelessness, and drug use (Pollack, 2009)—conditions specifically named as intimidating or unsafe by participants in this research. For some, this meant prioritizing immediate safety over accessing services that might support their long-term resettlement.

Another participant, a white woman in her late 40s named Kim, held a different perspective. She felt energized by the street, even when it was dangerous. “There *are* challenges [to sex work], but I feel free.” After years of this work, Kim knew she must be cautious and observant to survive—sometimes “acting paranoid” about the dangers she faced. But these conditions were, at times, dynamic and exciting:

it’s a bit of a thrill . . . just you know *getting through* day after day . . . like oh there’s danger over there, okay so now I’m gonna go over here and figure out what I need to do to survive. I’m zig zagging away from monsters.

Here, Kim’s outlook illustrates how habitus operates: “continuously transform[ing] necessities into strategies, constraints into preferences, and, without any mechanical determination, it generates the set of ‘choices’ constituting life-styles” (Bourdieu, 1984: 175).

These preferences also structured Kim’s resettlement goals; she was explicitly disinterested in a life she perceived as conventional. “I don’t think I would do well with a 9 to 5 . . . it’s so restrictive, you’d live your life in a box,” she said shaking her head. Critiquing the focus of resettlement organizations, Kim explains, “They just want to rein you in . . . pacify you. You get your 40 hours at McDonald’s and you’re supposed to be thrilled . . . like that’s some best-case scenario for someone with a record. Absolutely not happening.” Here, Kim laid bare some of the normative assumptions resettlement organizations make about the “help” criminalized women are thought to need and the kinds of aspirations deemed realistic (Quinn, 2020). For Kim, and others included in this research, such assumptions were at odds with the life they desired and thus served to discourage their participation in these services.

Throughout this section, I have illustrated how criminalized women have long mobilized vigilant dispositions to keep themselves safe. Although criminalized men are also vigilant about their environments and relationships, existing research has firmly anchored these dispositions in specific experiences of incarceration (e.g. walking the yard, avoiding violence in prison). In contrast and for the criminalized women included in this research, their embodied concerns about safety blurred the distinction between community and carceral that have defined men’s vigilance. The women included in this study relied on embodied dispositions to keep themselves safe long before their experiences of incarceration, destabilizing the focus on the isolated effects of imprisonment. Rather than ensuring their safety by violent posturing as men are suggested to (e.g. Caputo-Levine, 2013; Page and Goodman, 2018), criminalized women displayed a heightened awareness of their vulnerability and developed a set of intuitive “rules” or protective dispositions in response. They moved quickly through public spaces, generalized widely from unsafe experiences (e.g. avoided certain types of men), exercised vigilance about their environments and the body language of others, accepted the volatility of their lives, and (for some) thrived amid opportunities for change and adaptation. However, as is the case for men (e.g. Caputo-Levine, 2013; Martin, 2018), criminalized women’s adaptations were often at odds with resettlement programming goals. This tension resulted in criminalized women’s skepticism of “helping” efforts and resistance to accessing services offered by resettlement organizations, which sometimes further entrenched their social marginalization and the unsafe conditions they faced.

## Conclusion

By extending existing research on carceral habitus and resettlement using feminist considerations of transcarceration, this article has accounted for the constellation of embodied and adaptive dispositions accrued by criminalized women within, beyond, and prior to their imprisonment. Instead of mobilizing criminalized women’s trauma and backgrounds as “risk factors” for their criminalization and/or recidivism (for critique, see Davidson and Chesney-Lind, 2009), this article has shown how these experiences are brought to bear in daily life throughout women’s resettlement—documenting *how* these experiences matter, *when*, and *for whom* (also see Crewe, 2015; Walklate, 2011). For the women included in this research, their habitus (i.e. dispositions, expectations, preferences) was structured *by* and structuring *of* their transcarceral experiences of marginalization, isolation, and frequent threats to their safety. As the concept of habitus denotes, participants came to anticipate continuity in their experiences and moved through the world accordingly (e.g. moving quickly through public spaces, generalizing widely from unsafe experiences, vigilance about their environments and the body language of others, being resistant to forming social connections, accepting the volatility of their lives, and learning to thrive amid instability). Throughout their lives, these intuitions and adaptations sedimented, structuring the boundaries of what was imagined as possible and desirable for their relationships and resettlement.

The aim in highlighting embodiment was not to individualize these experiences, but to make connections between criminalized women’s dispositions and wider social structure. The conceptual resources available in Bourdieu’s theoretical work on habitus are especially adept at illuminating these linkages. In particular, by expanding the view of criminalized women’s experiences—especially their habitual and embodied dimensions—this analysis is able to be more faithful to the adaptive quality of their decisions and dispositions, making space to honor their creative routes to survival and the strategies that kept them safe amid challenging conditions throughout their lives. From this emerges a subject that is not only known by her criminalization, but also by her creativity, resourcefulness, adaptability, and resilience (Walklate, 2011; Western, 2018).

There remain considerable opportunities for future research on the intersections of transcarceration and habitus for criminalized women, some of which are indicated by limitations in my data. For example, the women included in this research experienced short and serial incarceration—a trajectory which has been described as “uniquely destabilizing” as individuals become caught in “perpetual cycles of ‘pre-incarceration’ and ‘re-incarceration’” (Comfort, 2016: 63, 66). Although existing research on carceral habitus has relied on the contrast between “inside” and “outside” (e.g. Martin, 2018), my research has illustrated that for criminalized women (who typically serve very short sentences) such boundaries are blurred, unstable, and, in their own estimations, often unimportant for their experiences of resettlement. Instead, their primary focus throughout this process remained on the social isolation and threats to their safety that were continuous features of their social realities. Given this observation, additional research is required on women serving long sentences and men serving short sentences to further disentangle the gendered habitus and resettlement pathways illuminated here.

In addition, the majority of the women included in this research also did not have children or were not (and not intending to be) their children’s primary caregivers. Although atypical, this feature enabled my analysis to shed light on the overlooked experiences of those women for whom children cannot or do not act as “turning points” in their resettlement (cf. Harding et al., 2019). However, because motherhood is an embodied experience and pro-social resource for many women, future research is required to add further dimensions to the (trans)carceral habitus that can account for women’s caretaking responsibilities and/or aspirations. Finally, my data primarily speak to the kinds of dispositions that were shared across women from different racial/ethnic backgrounds, with some distinctions (mainly of degree) that highlighted the specific and situated experiences of Indigenous women in Canada. Further work is required to clarify the intersectional inflections of the (trans)carceral habitus as a theoretical concept, particularly for women of color.

In addition to providing a more nuanced understanding of criminalized women’s lived (and embodied) experiences of exiting prison, these findings may also be used to better inform the rehabilitative interventions and desistance-focused projects of identity reconstruction offered by prison and probation staff (Burnett and McNeill, 2005), community corrections (Haney, 2010), and volunteers and voluntary sector practitioners (Quinn, 2020). In particular, criminalized women’s formative experiences and traumas may encourage (adaptive) dispositions that can complicate (or reduce) their willingness to access resettlement services (McKendy and Ricciardelli, 2020). For example, because of their lifelong experiences of social marginalization, the women included in this research were skeptical of others and quick to make assumptions of ill-intent; unsure, nervous, and easily put off within interactions with helping professionals; wary of disclosing personal information; inconsistent in their attendance of scheduled resettlement programming; and sometimes explicitly disinterested in the normative conceptions of “rehabilitation” that motivated resettlement organizations and their practitioners. A greater knowledge and understanding of these dispositions may inform better service provision by assisting helping professionals anticipate the impact and interpretation of their rehabilitative interventions (e.g. Walklate, 2011), including at the pre-conscious level.

Given criminalized women’s (trans)carceral habitus, certain rules and policies commonly utilized elsewhere (e.g. strict attendance, rotating staff/volunteers, initial information gathering, normative assumptions about the “help” ex-prisoners want or need) may be especially detrimental for their participation in resettlement programming. Instead, a greater appreciation of the way that (helping) relationships are experienced would mean a more patient, flexible, and forthright approach that gives criminalized women the space and opportunity to adjust their expectations and habitual responses. In addition, there may be a benefit in building in particular activities or demonstrations intended to fortify trust—as Sarah’s gesture did for Ofelia—within resettlement programming agendas. However, attending to the (trans)carceral habitus has also illuminated a huge challenge to, and for, such resettlement efforts. Criminalized women’s habitus, their “feel for the game” (Bourdieu, 1998: 25), and the embodied strategies they have long relied on to navigate danger and disappointment is, in many cases, precisely what is keeping them alive. This raises ethical questions about trying to alter these protective dispositions. Many “rehabilitation” efforts and resettlement programs are explicitly premised “on the assumption that an individual’s behavior can be targeted to eliminate the risk they pose to self or others” (Pollack, 2009: 85). Yet, based on this research, it remains uncertain whether or not these kinds of resettlement programs and tools could ever offer criminalized women “better” dispositions, expectations, and preferences for navigating the transcarceral marginalization and dangers they habitually face.

## Notes

1. This project focused on women’s experiences of exiting prison and sought to capture the lived realities of resettlement, especially the role of social ties in this process.

2. Prior to entering the field as a researcher, I volunteered as mentor at a local women’s prison, a legal resource to women in court, a participant in restorative justice sharing circles, and a receptionist at a resettlement organization for women. These experiences helped me to establish myself as a known regular who was visible and perceived as legitimate within this environment (Browne and McBride, 2015). This not only assisted in my participant recruitment, but also served to mitigate potential concerns about an observer effect as the majority of participants had known (or at least seen) me for months prior to choosing to take part in this research.

3. Such a response—of keeping others at bay because relationships were experienced as painful or hostile—might also be described in the psychoanalytical literature as a “defense mechanism” (Cramer, 2000). The difference here, and for Bourdieu, is the tight connection made between an individual’s place in social structure and their habitus rather than positing a relationship between past experiences and personality structures.
